# The Evolution and Sustainability of Environmental Health Services in the Azraq Refugee Camp, Jordan: A Qualitative Study

**DOI:** 10.3390/su16177758

**Published:** 2024-09-06

**Authors:** Nikki Behnke, Brandie Banner Shackelford, Amjad Dawood, Rachel A. Oommen, Raymond Tu, Marielle Snel, Iyad Al Samawi, Samer Talozi, Darcy Anderson, Ryan Cronk, Jamie Bartram

**Affiliations:** 1 The Water Institute, The University of North Carolina at Chapel Hill, Chapel Hill, NC 27516, USA; 2 Center for Water Security, Sanitation, and Hygiene, United States Agency for International Development (USAID), Washington, DC 20523, USA; 3 United Nations High Commissioner for Refugees (UNHCR), Lilongwe P.O. Box 30230, Malawi; 4 World Vision, Amman 11547, Jordan; 5 Department of City and Regional Planning, The University of North Carolina at Chapel Hill, Chapel Hill, NC 27516, USA; 6 Schulich School of Medicine and Dentistry, The University of Western Ontario, London, ON N6A 3K7, Canada; 7 Save the Children International, Amman 11191, Jordan; 8 Civil Engineering Department, Jordan University of Science and Technology, Irbid 3030, Jordan; 9 School of Civil Engineering, University of Leeds, Leeds LS2 9JT, UK

**Keywords:** refugees, Syria, Jordan, Middle East, water, sanitation, hygiene, WaSH, solid waste management, sustainability

## Abstract

The Syrian civil war displaced more than half of the Syrian population, including over 660,000 registered refugees who fled to neighboring Jordan. Environmental health services (e.g., water, sanitation, hygiene, and solid waste management) are critical for refugee health. Still, they may strain resources in host communities and must evolve in protracted crises. We studied environmental health services in the Azraq refugee camp in Jordan to identify the stakeholders and their roles in service provision, assess stakeholder communication and coordination, and evaluate sustainability. We conducted 25 interviews with stakeholders involved in environmental health service provision. We found that non-governmental and United Nations organizations had well-defined responsibilities, but the roles of donors, the Jordanian government, refugees, and the host community needed clarification. Conflicting standards and mismatched donor expectations with on-the-ground needs sometimes created challenges for coordinated and efficient service provision. The basic needs of refugees were generally met and services improved somewhat over time, but political obstacles and inadequate resources complicated the path toward sustainable services. Early incorporation of sustainability in planning and increased efforts to build the capacity of refugees to contribute and take ownership of environmental health services will likely enhance long-term environmental health service provision and development outcomes.

## 1. Introduction

Access to adequate water, sanitation, and hygiene (WaSH) services is a human right and should not be denied based on immigration status [1]. However, refugee influxes can cause tensions with host communities by pressuring natural resources, reducing water availability and quality, and contributing to other forms of environmental degradation [2–5]. Policy approaches to WaSH service provision for displaced populations are often not designed for protracted crises [6,7], and challenges related to resource scarcity, politics, and governance impede progress toward sustainable service provision in such settings [4,8]. The Syrian civil war has resulted in one of the largest refugee crises since World War II, with 7.2 million Syrians displaced to neighboring countries. As of October 2023, Jordan hosts over 660,000 registered Syrian refugees [9]. However, Jordan is one of the world’s most water-scarce countries and providing WaSH services to a large refugee population long term is challenging [10].

Before the Syrian refugee crisis, Jordan was already facing challenges with water scarcity. Influxes of refugees have increased demand for groundwater resources, accelerated groundwater depletion [11], and highlighted underlying challenges with water service delivery [11]. For example, in the northern governorates near the Syrian border—where many refugees reside—water usage has increased. Hydrogeological studies indicate over-pumping, with draw-down rates of aquifers exceeding recharge rates in the vicinity of camps [12]. The water available for domestic use decreased substantially between 2011 and 2016, from 88 to 66 L per capita per day [13]. This issue is of growing concern to refugees and the host community [14,15], leading to tensions. For example, some have described refugees as “thieves” who are taking natural resources to which they are not entitled [16]. Public polling data indicate that some among the Jordanian public perceive refugees to be responsible for reducing water availability, increasing solid waste, and degrading groundwater quality due to lack of sanitation [17]. In some cases, riots and violence have broken out due to water supply challenges [16–19]. Responding to this crisis is complicated by political discourse, which frames the arrival of refugees as a detrimental strain on national resources [11].

Nevertheless, as this crisis persists, the Jordanian government, United Nations (UN) agencies, and non-governmental organizations (NGOs) have worked to develop more sustainable WaSH services for Syrian refugees. Resource scarcity and coordination have prevented progress [20,21]. Camps initially established to house refugees were primarily established ad hoc, with limited opportunity to plan WaSH service provision before the arrival of inhabitants. However, as these camps reached capacity and the Syrian civil war continued, the Jordanian government and partner organizations sought to plan the next camp. Azraq opened in 2014 after Jordan’s largest Syrian refugee camp—Za’atari—reached capacity. It was built to incorporate “lessons learned” from the more hastily formed Za’atari and is considered to be one of the world’s “best-planned” refugee camps. For example, Azraq was planned using a bottom-up approach. Housing units are grouped into “villages” that provide cooking, cleaning, bathing, and sanitation areas. Residents can request placements next to family members and friends, compared to random assignment, which is more typical in other camps and is intended to preserve the traditional social arrangements of Syrian communities. Housing and other structures are constructed with more durable materials to better withstand and protect against desert storms [22,23]. This type of planning is rare among refugee camps and studying it can provide insight into how to incorporate sustainability in WaSH and related environmental health services for displaced populations.

We undertook a qualitative case study of WaSH and solid waste management services (hereafter “environmental health services”) in the Azraq refugee camp. We used semi-structured interviews with NGO and UN staff and refugees in environmental health service provision. Our objective was to understand the implementation, evolution, and sustainability of camp services. We defined sustainability as the long-term ability to operate and maintain services such that they continue to serve the target population in the future. In the context of the Azraq camp, we examined sustainability considering the protracted nature of the Syrian refugee crisis and the likely need to continue to provide services to the refugee population for the foreseeable future as part of a permanent or semi-permanent service delivery model.

We asked three research questions: (1) What stakeholders are involved in environmental health services, and what are their respective roles? (2) How do stakeholders coordinate and communicate regarding environmental health services? (3) How do participants perceive camp sustainability and change in environmental health services over time?

## 2. Methods

### 2.1. Study Setting

The Azraq refugee camp is Jordan’s second-largest camp for Syrian refugees, with approximately 40,000 residents [24]. The camp is co-managed by the UN High Commissioner for Refugees (UNHCR) and the Syrian Refugee Affairs Directorate, a department of the Jordanian government. It is in a remote area of the Jordanian desert, approximately 120 km east of Amman and 20 km from the nearest town, Azraq.

The camp contains identical white steel shelters that are arranged into blocks within “villages”, which is an approach intended to create a “greater sense of ownership and community among residents” [25]. Large families occupy adjacent shelters, and villages are organized by Syrians’ towns of origin [22]. Employment opportunities in the camp are scarce, though NGOs implement an “incentive-based volunteer” program in which refugees are paid hourly for temporary work on NGO projects [26].

Two boreholes pump water to camp residents through pipes connected to communal tap stands. The UNHCR reports that 302 tap stands (each with four faucets) distributed an average of 42 L per capita per day in December 2018 [27]. Water quality is monitored by NGOs in the camp, based on Sphere and Jordanian government standards [28,29]. Sanitation blocks, which include a latrine and a shower, are gender separated. Two sanitation blocks—one for each gender—are provided for every 12 shelters [30]. Azraq is among the first refugee camps in the world to have an on-site wastewater treatment plant [23]. NGOs conduct hygiene promotion programs and routine solid waste collection [27,31].

### 2.2. Sampling

This research was conducted as part of a partnership between the Water Institute at the University of North Carolina at Chapel Hill, a research institution, and World Vision, an NGO in the Azraq camp. World Vision and their contacts within other NGOs identified study participants with relevant knowledge and roles in environmental health services, specifically related to the implementation of WaSH and solid waste management service delivery, stakeholder roles and responsibilities, changes in service delivery over time, and barriers and facilitators to sustainability. Identified individuals included roles such as engineers, WASH officers, behavior change specialists and hygiene promoters, project managers, community mobilizers, and director-level positions. We then purposively sampled from among these suggested individuals to achieve representation across job roles and responsibilities, backgrounds, and experiences. This included participants across different job functions (including monitoring and evaluation, grants management, finance, and project management), disciplines (including behavior change communication, WASH engineering, hygiene promotion, solid waste management, and community mobilization), and professional level (including both supervisors, supervisees, and NGO staff at the coordinator, consultant, project manager, director, and other levels). The full list of individuals is provided in the Supplementary Materials, File S1.

Due to research activity restrictions in the camp, we were only permitted to interview people affiliated with an NGO or the UN. Our sample included refugees, but only those working for NGOs in some capacity, such as incentive-based volunteers for NGOs or community leaders.

### 2.3. Data Collection

We collected data from May to July 2018. We used semi-structured interviews, as this qualitative method is well suited to capture lived experiences, assess complex roles and relationships between people and organizations, and describe changes over time in different factors related to sustainability [32]. We developed a semi-structured interview guide with questions on the following topics: stakeholders’ roles and perceptions of their responsibilities, successes, and challenges; communication and information sharing processes; coordination processes; baseline and current environmental health services and reflections on how and why they have changed over time; and perspectives on the extent to which Azraq is sustainable and opportunities to improve sustainability. We asked how different stakeholders address sustainability within the camp. We probed on how participants self-defined sustainability within their work context and how sustainability was incorporated. We developed the initial guide in English and then translated it into Arabic.

Two authors (NB and BBS) conducted interviews in the Azraq camp and NGO headquarters offices in Amman. Interviews were conducted in English or Arabic, following the participant’s preference. The lead author is a native English speaker and has some Arabic proficiency. For Arabic interviews, an interpreter assisted with translation where necessary. To minimize potential bias, data collectors indicated to participants that they were not affiliated with any of the implementers or regulators within the camp and that information would be anonymized and used exclusively for research purposes.

Interviews lasted, on average, 35–40 min and were audio-recorded when participants gave permission (*n* = 24, 96%). All recordings were transcribed and translated into English as necessary for analysis. For non-recorded interviews, we used detailed notes compiled during and immediately after the interview. The interview guide is included in the Supplementary Materials, File S2.

### 2.4. Analysis

Three coders (NB, RT, and AO) analyzed English transcriptions using Dedoose (version 8.1), a qualitative analysis software. Two rounds of coding were conducted; in the first round, a codebook was developed to capture critical themes in the data. The team met weekly to discuss potential new codes and adjust the codebook accordingly. Once the team had reached a consensus, the codebook was finalized. Once the first round was complete, the original codes were deleted from transcripts, and a second round of coding was conducted using the finalized codebook. Researchers examined code co-occurrences and used thematic analysis to identify trends in the data.

### 2.5. Ethics

This study was approved by the Institutional Review Board of the University of North Carolina at Chapel Hill (18–0922). We obtained a permit from the Syrian Refugee Affairs Directorate to enter the camp for this research. Each participant provided written informed consent in either English or Arabic.

## 3. Results

### 3.1. Study Sample

The study sample comprised 25 semi-structured interviews with staff from NGOs and UN agencies, refugee incentive-based volunteers, and refugee community leaders (Table 1). The full listing of job titles for participants is provided in the Supplementary Materials, File S1.

### 3.2. Stakeholders, Roles, and Responsibilities

We identified stakeholders and their roles and responsibilities related to environmental health services in Azraq. During coding, we identified five stakeholder groups: implementers (NGOs, UN agencies, bilateral aid agencies, and private contractors), funders, Jordanian government ministries, refugees, and the host community.

#### 3.2.1. Implementers

Implementers were responsible for day-to-day activities to deliver environmental health services. Responsibilities among implementers were clearly defined, with minimal overlap between stakeholders (Table 2). Implementing stakeholders often released literature describing their roles in the camp’s environmental health services, though the information about the coordination of specific activities was piecemeal and often incomplete. While technical support was available to implementers, the precise roles and responsibilities were not always clear. One NGO staff member described these roles and responsibilities as follows:

“Anything that is about relationship with the donor, changing anything to the design, in terms of changes to the contract, there, we would always go to the support office. Then, to the regional office more for if we need additional technical support, or in terms of getting support for any evaluation or evidence, that’s usually the regional office. But those lines are not always 100% clear.”

Roles and responsibilities for stakeholders remained relatively consistent for the first 24 months of camp planning and operation, with some turnover in responsibilities happening after. Participants described handing over responsibilities from one NGO to another as challenging. For example, one NGO staff member explained that they had continued working on WaSH services for more than two months after their contract ended, waiting to halt operations until the new implementing NGO was approved.

#### 3.2.2. Funders

Funders were not involved in day-to-day operations, but participants described them as profoundly influencing the strategic direction of environmental health services in the camp. For example, one NGO staff member explained how donors had become more interested in sustainability as the refugee crisis has persisted:

“This is the first question everyone would ask us, ‘Yes, sustainable. You have existing strategy for your project? How do you make sure that this project will be carried forward in the future? What factors do you think it’s achieving, and who is from the community or from Syrian refugees who will take it forward and the responsibility afterward?’ You need to assign it even in the proposal phase, so they make sure there’s a plan ahead.”

#### 3.2.3. Jordanian Government Ministries

Jordanian government ministries were primarily responsible for regulating and monitoring environmental health services. We identified five ministries and one directorate specifically created to manage Syrian refugees: the Ministry of Environment, the Ministry of Health, the Ministry of Water and Irrigation, the Ministry of Planning and International Cooperation, the Ministry of Municipal Affairs, and the Syrian Refugee Affairs Directorate.

The Ministry of Health and the Ministry of Environment’s primary responsibilities were establishing and monitoring water quality standards. The Ministry of Water and Irrigation tracked water consumption within and outside the camp relative to the region’s available water supply. The Ministry of Planning and International Cooperation was primarily a regulatory body whose approval was necessary to plan activities in Azraq. The Ministry of Municipal Affairs did not work in the camp at the time of this study but was responsible for waste management in the nearby town of Azraq and, therefore, engaged in discussions about possible future expansion of the camp. The Syrian Refugee Affairs Directorate was responsible for camp management and security and had to approve any activities in the camp.

#### 3.2.4. Refugees

Refugees were involved in environmental health services as beneficiaries, WaSH committee members, and incentive-based volunteers. Participants described the importance of refugee feedback on program design, including infrastructure and adopting behaviors like handwashing or recycling. Refugees participated as unpaid volunteers in WaSH committees, which NGOs, the Syrian Refugee Affairs Directorate, and camp management oversaw. Committees met monthly or every two weeks and most attendees were women. WaSH committees were responsible for essential operation and maintenance tasks, as described in the following account:

“The WaSH committee was responsible for the operation and the maintenance of the WaSH facilities in their blocks. We provide them with basic tools so they can do their own maintenance for the toilets, and they help us in the hygiene promotion activities like committee mobilization and help us prepare the events, et cetera.”

Incentive-based volunteers were paid an hourly wage for their work and were hired through a livelihoods program run by NGOs. The incentive-based volunteers interviewed for this study worked in solid waste management by sorting waste for recycling or as community mobilizers to raise awareness about proper waste disposal. In some cases, incentive-based volunteers described having professional experience from when they lived in Syria that was relevant to their role in the camp. NGO staff described the incentive-based volunteer involvement in engaging refugees in environmental health services and providing livelihood opportunities and dignity.

We did not identify any informal refugee institutions; participants explained that the camp’s highly controlled environment meant that refugee groups or institutions could not form grassroots organizations.

#### 3.2.5. Host Community

The host community was isolated from the Azraq camp, and participants believed this was intentional to ensure minimal contact. One NGO staff member described how he thought that the relationship with the host community, which views Azraq camp as separate, is isolated from their community:

“Azraq camp is very isolated from anybody and no CBO would be interested in doing anything inside because it’s community-based because they’re all caring about their community.”

However, the host community was cited as an essential stakeholder in two ways. First, the camp had environmental impacts that extended beyond the camp’s boundaries, particularly about the shared water supply and issues of water scarcity. Second, the host community was considered a potential partner for future environmental health service provision.

### 3.3. Stakeholder Coordination and Communication

We identified three key stakeholder and communication themes: official coordination and communication channels, competition for funds, and environmental health standards and guidelines.

#### 3.3.1. Official Coordination and Communication Channels

Among implementers in the camp, NGOs involved in environmental health services were part of a WaSH working group, which met every two weeks. At the time of this study, these meetings were led by two UN agencies (UNHCR and UNICEF), with some other implementers—but not all—attending. Sessions covered updates, plans, challenges, and expectations. Participants described email, phone, and in-person communication between the various NGOs and UN agencies in the camp. Participants considered the amount of contact between NGOs involved in environmental health services sufficient to facilitate effective coordination. However, given the large number of actors in the camp, communication was sometimes slow. One NGO staff member described how smaller groups dedicated to WaSH issues had been formed to streamline communication and coordination:

“When it comes to making some decisions, it’s sometimes slow because if it involves 19 or 20-something NGOs working in the camp, that can be difficult. If it’s in the WASH cluster, because it involves fewer actors, it’s faster and more efficient. Faster, from the perspective of, they just agree on the meeting, the WASH Sector Meeting, and then we go ahead. The bigger coordination that’s a little difficult because you need to engage different actors, and… not all of them can make the decision right away. Some of them have to get back to headquarters, et cetera.”

Participants described three primary mechanisms for communication between refugees and the NGOs: a hotline, feedback sessions, and word of mouth through community leaders. The hotline was a platform for refugees to report problems with environmental health services. It connected them to an incentive-based volunteer, who directed the information to the relevant NGO. NGOs put up posters with information about how to reach the hotline. One NGO facilitated weekly feedback sessions to offer refugees an opportunity to bring concerns to representatives from each NGO. The meetings were used for NGOs to give updates and for refugees to ask questions or provide feedback. Community leaders played an important role in facilitating communication. One leader described himself as the voice of the refugees:

“[I am] the voice of the refugees in this village, and [I am] in direct contact with the NGOs… to ask them for their needs for the village and this block.”

NGO staff held meetings with community leaders, who were elected to represent their respective villages and blocks. NGOs relied upon community leaders, including WaSH committee members, to deliver messages about environmental health services throughout the camp.

#### 3.3.2. Competition for Funding

Some perceived competition for funding to be a barrier to coordination. Although NGO staff reported having strong working relationships with other NGOs, participants discussed the challenges they faced in coordination with other NGOs in an environment of diminishing funding. One NGO staff member explained the challenges as follows:

“There’s always that kind of phenomenon that—all the NGOs—somehow they compete for the same funds. … In the end, everyone wants to make sure that they don’t share everything and that they keep their donor and their funding. I think the whole nature of the setup of this industry… by nature doesn’t really allow to have 100% efficient coordination.”

Competition for resources did have some positive impacts. Several participants noted that, as funding becomes scarce, donors become more interested in sustainability. Others noted that as there were fewer funding opportunities, funders could demand more of the projects they supported. However, some participants expressed frustration with having to cater to funder interests. One NGO staff member noted that eight years into the Syrian crisis, it was easier to obtain funding from donors if the project involved an innovative idea or technology.

#### 3.3.3. Environmental Health Guidelines and Standards

Environmental health guidelines and standards were necessary for informing implementers’ activities. However, different agencies had different standards for WaSH, solid waste management, and other issues, often perceived as conflicting. One NGO staff member described how Sphere, the Jordanian government, and the various NGOs all had standards:

“It’s very complicated. Sometimes, when we’re dealing with other NGOs, we get two standards. How to standardize everything took a lot of effort and a lot of talks. Sometimes both they’re right but according to their standards.”

Other NGOs confirmed this. Of the eleven participants who mentioned using environmental health guidelines and standards, there needed to be more consistency, and some participants reported using multiple guidelines and standards simultaneously: six used Sphere guidelines, four used Jordanian government standards, and six used NGO-specific guidelines.

In some instances, international standards such as Sphere were needed to capture the cultural context of the camp adequately. These standards were focused on quantitative metrics such as liters of water per person but did not consider cultural factors. For example, the central latrine blocks with shower facilities that were initially constructed met the Sphere Standards but did not align with refugees’ bathing preferences and gender norms around privacy. As described by one NGO staff member:

“Even we used to construct shower facilities, but the women didn’t use those shower facilities because [of] the culture protection perspective. So, we are providing the minimal standards, but because of the culture… some men, didn’t allow his wife to go to those shower facilities. So sometimes we need to sit we the community, to talk with the people and what’s the best practice, and how we can serve you.”

## 4. Sustainability and Change over Time

We assessed changes in environmental health services since the opening of the camp and identified three key themes related to sustainability: perceived permanence, ownership and responsibility, and financing.

### 4.1. Change in Environmental Health Services

Participants reported that service levels had generally improved over time. For example, the camp initially relied upon water trucking, but NGOs had drilled boreholes to make water delivery more cost-effective and sustainable. Improvements were particularly notable regarding cultural sensitivity. The original design of sanitation blocks was not tailored to refugee preferences but had been updated to be more culturally appropriate based on refugee feedback. For example, toilets were initially installed with “Western-style” sitting toilets, which were later converted to “Arab-style” squat toilets:

“We installed it exactly as it was drawn. It was designed because there was nobody there like refugees or beneficiaries that we can take the feedback. But afterwards, when there was beneficiaries, we started to get in the feedback… we redesigned the hole and the septic tank to be very appropriate to the culture of the beneficiaries, installed the Arabic seats.”

NGOs had begun constructing greywater systems in the camp, improving the drainage from shelters, where many women bathe due to cultural sensitivities around public showers. Some participants pointed to improvements in services—such as the incorporation of recycling and the possibility of composting in the future—as sustainability indicators.

Other participants suggested that more sustainable service delivery required that implementing organizations focus more on hygiene promotion activities, participatory decision-making, and behavior change instead of infrastructure projects alone.

Some declining service levels were reported; most notably, a decrease in water pressure, a decline in the amount of time when water is available each day, and an increase in crowding around water points were discussed. These declining service levels were generally thought to have resulted from camp population growth. Water conservation and challenges with scarcity were key concerns and challenges for sustainability.

### 4.2. Perceived Permanence

Another change described by participants was the perceived longevity of the camp. Participants described a gradual shift in thinking from emergency response to longer-term development. One NGO staff member noted the following:

“At the beginning, we were not looking for sustainability; we were just responding to an emergency. Then, with time, you realize that it takes not days or weeks or months, it takes several years… the thinking gradually started moving to sustainable solutions, sustainable approaches.”

One NGO staff member noted that this shift in thinking mirrored the political situation in Syria, not just in Azraq but in refugee response efforts more broadly:

“In Za’atari in 2013, the thinking of sustainability was not there. You need to link it with the political situation inside Syria. When people started to believe that there is no political solution for their crisis, they started thinking about sustainable solutions for the host communities.”

Some actions that were taken to recognize the need for sustainable services included adapting infrastructure to better suit the cultural needs and preferences of refugees. For example, adapting toilet sit- to squat-style toilets and strengthening the privacy of bathing facilities to address concerns about women’s modesty. However, structural challenges were more difficult to address, either due to decisions about the camp’s location and lack of surrounding amenities, or low political will. Specifically, respondents questioned the possibility of sustainability of the Azraq camp due to a variety of factors, including the camp’s isolated nature, the dependence of refugees on NGOs and foreign aid, the lack of willingness of the Jordanian government to pursue sustainable solutions for the camp, and the lack of income-generating activities for refugees. One NGO staff member explained this issue as follows:

“We cannot talk about sustainability perspectives in the camp setting because [the] camp itself is not a sustainable structure. It is a temporary solution for refugees… We’re dealing with a protracted kind of humanitarian response in that country that has elements of development. But it still is not acute emergency stage anymore, but still those people are heavily dependent on international aid.”

### 4.3. Ownership and Responsibility

In reflecting on sustainability, participants frequently mentioned themes of ownership and responsibility, which were closely linked to financial issues. Some participants perceived that to achieve sustainability, responsibility for environmental health service provision would need to be transferred to the Jordanian government or the municipality of Azraq. Under this scenario, participants believed that refugees would need to be allowed to earn an income and contribute to municipal taxes. One NGO staff member explained this scenario as follows:

“The full sustainability will be, for me, if the municipalities take over the solid waste management in the camp… it usually only works if--the population through taxes from which we can pay the fees such as for municipal services such as solid waste management.”

Another NGO staff member explained that if the municipal government were to get involved, Azraq “would be as a part of like any town in Jordan.” However, participants acknowledged that political and financial obstacles impede this possibility.

Other participants echoed this shift towards sustainability and mentioned that ownership—and refugee participation—had become more significant priorities. Another critical change that NGO staff noted was refugees’ willingness to voice their concerns and give feedback. One NGO staff member suggested that refugees learned their rights and became more comfortable speaking up because of participating in WaSH committees and other refugee groups.

Social elements of sustainability were frequently addressed, with respondents noting the importance of promoting ownership among refugees, empowering WaSH committees, and focusing on hygiene promotion and behavior change. Ownership was cited as a particularly challenging concept in Azraq due to the isolated nature of the camp. One NGO staff member, when discussing ownership, stated the following:

“At the same time, the ownership of what? …They are, let’s say, waiting when the war will be over and they will go back. They don’t have an attachment to that specific area as their own.”

Some participants made recommendations to develop a sense of ownership among refugees. One NGO staff member suggested inviting refugees to the WaSH working group meetings or engaging them in day-to-day operations and maintenance:

“I felt we need to empower the WaSH committees more. We need to give them more responsibilities. We’re doing much more than we should in terms of operation and maintenance because we’re doing the maintenance itself for all the blocks. If we find a door broken, we will fix it, but I don’t think some NGOs want to do these small things.”

### 4.4. Financing

Several participants discussed the importance of reducing dependence upon NGO funding for camp services. This included promoting the use of incentive-based volunteers in projects, collecting token contributions from refugees, improving the cost-effectiveness and efficiency of service delivery, and seeking ways to increase the return on investment for specific projects, such as selling recycled materials to buyers outside of the camp.

However, some participants questioned the feasibility of these ideas. The distance of the camp from urban centers increased costs such as transportation, and regulations sometimes could have improved efficiency and revenue generation. One NGO worker noted that solid waste management was unlikely to ever recover costs from selling recyclables and that funds would always be needed to pay for the service:

“It is impossible to have a sustainable model for the solid waste project since the income will never cover the expenses. There should be a fund to cover these services. The market for recyclable materials in Jordan is poor. The location of Azraq–it is far away. The transportation cost is the highest. There are also many government restrictions for implementing any project such as composting.”

## 5. Discussion

We conducted a qualitative study of environmental conditions in the Azraq refugee camp, examining (1) stakeholders and their respective roles in environmental health service provision, (2) coordination and communication mechanisms between stakeholders, and (3) perceived sustainability of the camp and changes in environmental conditions over time. We discuss our findings in the context of the prior literature below.

### 5.1. Stakeholder Roles

We found that various governments, NGOs, multilateral organizations, and bilateral agencies were involved in environmental health service provision. However, their roles and responsibilities were typically well defined and non-overlapping. The division of responsibilities among different implementing stakeholders in Azraq was consistent with international humanitarian response guidelines; clearly defined roles can minimize duplication of efforts and maximize efficiency [7,25,33]. Prior studies in Jordanian refugee camps suggest that understanding these management dynamics is critical for ensuring that wastewater treatment plants are operational, efficient, and environmentally sustainable [34,35]. Our findings add to this body of literature by documenting stakeholder roles among a broader range of environmental health services.

Refugees were engaged in environmental health service provision in formal roles as incentive-based volunteers for NGOs. Still, we found no informal institutions related to environmental health service provision in the Azraq camp. Prior research in refugee camps in Sudan suggests that community leaders and committees are a highly effective way to manage WaSH systems and ensure sustainability [28,29]. Informal institutions have been found to aid resource governance in other settings of informal settlements and urban areas where refugees have settled [1,6,36]. In the Azraq context, leaders of WaSH committees formally approved and organized by camp authorities may help to fill some of this gap, as they have community representation.

We identified the host community as a stakeholder, even though they had little direct interaction with the camp. The host community was a key stakeholder primarily because of water usage and scarcity concerns. Participants’ concerns about the adverse impacts of the Azraq camp on the host community are consistent with the literature on health and environmental risks to host populations living near camps [37]. Moreover, increased water stress and tensions between refugees and host communities have been reported in other parts of Jordan [16,18]. Contributing to this tension is the perception that Syrian refugees are inefficient or wasteful with water use, being accustomed to the more water-rich environment they have fled in Syria. Public information campaigns have been launched to encourage water conservation for washing dishes, showering, and other behaviors [14]. Participants in our study recommended coordinating with the host community for the potential expansion of WaSH services, and prior studies support this recommendation as an important success factor in developing practical, sustainable environmental health services [38,39].

### 5.2. Coordination and Communication within the Camp

Participants generally found that communication between implementers needed to be improved to coordinate service provision, and the primary challenges were related to the scarcity of funding and differing standards and guidelines for environmental health services. The need to cater to donor expectations and preferences and to match donor priorities to conditions on the ground are commonly reported challenges for implementing organizations [40–43]. We found that participants’ perceptions of impacts on program quality were mixed; some participants perceived that donor expectations did not reflect the reality on the ground, while others perceived that funder expectations drove more sustainable programming.

Several participants discussed the need to adapt their programs to compete for funding, mainly as donor interest has dwindled since the beginning of the Syrian crisis. This is consistent with the findings on humanitarian cluster coordination, where competing for funds can lead to distrust and animosity between agencies working in a camp [43,44]. This is particularly challenging for NGOs working to implement longer-term programs and infrastructure. As support from the international community declines, stress on host country resources increases, and coordination suffers as NGOs compete for funding [44,45]. While the coordination and communication mechanisms facilitate the sharing of resources and information, competition over funds may lead some stakeholders to be less open about their planning and implementation.

We identified inconsistency in environmental health standards and guidelines applied by different stakeholders as a barrier to coordination. This consistency can lead to communication, clarity, and efficiency. A lack of harmonized standards for service delivery is a widespread challenge in both humanitarian response and international development [44,46]. The 2018 Sphere Standards and the UNHCR WaSH Manual both emphasize that WaSH indicators and standards should be aligned with national standards whenever possible in protracted situations, and their standards should be presented [7,29]. Different standards and guidelines offer the opportunity to incorporate different elements based on the stage and needs of a particular humanitarian crisis [45]. We did not find evidence that stakeholders had changed their standards and guidelines over time since the camp’s construction. However, this is a potential opportunity to harmonize efforts and plan for sustainability.

### 5.3. Political Climate and Ownership

The political climate around Syrian refugees in Jordan is complex. Jordan is not a signatory to the 1951 Refugee Convention or 1967 Optional Protocol, which defines standards for refugee treatment. A core principle of the convention forbids encouraging refugee repatriation in the event that they would face life-threatening conditions or constraints to personal freedom [47]. The government of Jordan maintains its position that the Azraq camp is temporary and not intended to be a long-term structure. Jordan encourages the return of refugees to Syria. Refugees are often referred to as “Arab brothers”, “irregular guests”, or “visitors”, implying their eventual departure [47].

Yet in the interim, the government of Jordan supports donor-led inclusion efforts as a temporary solution. For example, UNICEF and other international NGO partners planned (as of 2024) the construction of a new borehole and wastewater treatment plan. Considerable funding was also invested in WaSH infrastructure during the COVID-19 pandemic, particularly for hygiene [48].

### 5.4. Sustainability and Change over Time

We found that environmental health services in the camp had generally improved over time, particularly regarding cultural appropriateness to refugees’ needs [49]. However, water scarcity remains a persistent challenge, likely worsening over time [11,50–52]. Compared to the Sphere Standards, the water quantity per capita in Azraq exceeded the minimum recommended level of 15 L/person/day. However, this standard is based on basic survival needs, and the standards note that water quantity standards should be context-specific, with higher standards for protracted crises [29].

Resolving challenges related to water scarcity and environmental health service sustainability is linked to ownership and management of the camp and the need to transition to more permanent service delivery models that are better suited to protracted crises. As humanitarian crises transition from the emergency to the protracted phase, service delivery challenges and infrastructure needs change. Temporary infrastructure installed in the emergency phase is rarely intended to meet long-term demand and often suffers breakdowns and durability challenges. Governance systems in the emergency phase are established among organizations and agencies with experience and expertise in crisis response. However, these stakeholders often lack expertise or mandates for long-term service delivery [8,53,54].

High costs and low political will can deter establishing more permanent, durable infrastructure and governance systems. Participants in our sample who were refugees and NGO staff often expressed that transitioning the camp to government responsibility and management was the best solution for sustainability. This belief is consistent with prior research, which suggests that funders, NGOs, and multilateral agencies advocate for national governments to take responsibility but that governments themselves may be reluctant and desire a different division of responsibilities between government and non-government stakeholders [6]. Studies in Jordan and elsewhere have indicated unwillingness on behalf of governments to recognize the need for and invest in more permanent infrastructure that is better suited to the needs of protracted crises, in part because it recognizes and is perceived to incentivize long-term residency of refugees, who host communities may prefer to return to their home countries [6,11,14].

Ideas for revenue generation of environmental health services may make ownership more attractive. However, full-cost recovery is unlikely [55]. Solid waste management systems in Jordan have demonstrated 30–60% cost recovery [56,57]. Long-term operation costs of WaSH services in protracted humanitarian crises need to be better documented. Evidence from two refugee camps in sub-Saharan Africa suggests that water service alone costs USD 2–12 per refugee per year [58]. Costs in Azraq may be higher given its isolated location and water scarcity in the region and will need to consider the costs of sanitation and hygiene.

As existing donor funding dwindles, other funding sources will be needed to ensure sustainable environmental health service operation and maintenance. Some scholars argue that investment in sustainable environmental health services in refugee camps may benefit the host community if services are built to strengthen host communities when camps are no longer occupied [59]. Benefits to the host community may incentivize investment. However, given the remote location of the Azraq camp, opportunities to strengthen and integrate camp infrastructure into nearby municipal systems are less likely. Dissonant perspectives on sustainability and responsibilities can lead to clarity and an efficient allocation of resources [6]. However, in the case of the Jordanian government, this risk may be lower than in other settings. Despite reluctance to recognize the Azraq camp as a permanent structure, existing Jordanian policies suggest that sustainability is a priority for WaSH in refugee camps. The Jordanian government’s response plan for the Syrian crisis notes that developing large-scale WaSH infrastructure in refugee camps, including Azraq, is meant to increase the sustainability of WaSH services. The Jordanian refugee plan notes that environmental sustainability should be a “cross-cutting issue across all sectors and all interventions” [18]. More broadly, UNHCR’s WaSH Manual calls for stakeholders to prioritize and facilitate sustainability in their approach to WaSH in refugee settings [7]. Stakeholders are aligned in prioritizing sustainability, though neither document provides specific steps or recommendations to ensure sustainability is incorporated in WaSH programming in these contexts.

These plans and priorities reflect trends in refugee repatriation over recent years. The number of voluntary repatriations from Jordan documented by UNHCR peaked in 2019 at 29,400 and decreased to 4000 in 2022 [9]. Survey data from 2023 indicate that just 1% of refugees intend to return to Syria in the next 12 months [60]. While the Azraq camp was initially designed to be temporary, slowing trends in repatriation will likely eventually force the Jordanian government and camp authorities to confront challenges with infrastructure sustainability and permanence. The government of Jordan has taken some pre-emptive steps with large-scale investments in deeper wells, larger pipelines, and dam construction to address its water shortages. However, many of these investments were made prior to the Syrian refugee crisis, but there is widespread discourse among Jordanian media, politicians, and the general public that these investments will not be sufficient to cope with the additional burden of refugees [11]. Even without further influxes of refugees, aging infrastructure will experience more frequent breakdowns and require more extensive maintenance, raising the importance and urgency of planning for sustainability.

## 6. Limitations

Our sample comprised NGO, multilateral, and bilateral agencies working in the Azraq camp. Due to restrictions on research activities, we included refugees who were working for NGOs, for example, as incentive-based volunteers. Still, we were not permitted to recruit a broader sample of refugees or other stakeholders not affiliated with an NGO. These individuals may hold different perspectives about environmental health services, and their views are not represented in our study. Furthermore, while our informed consent process emphasized that results would be confidential, participants we were able to interview may have been reluctant to share certain sensitive information if it could be perceived to reflect poorly on them or their colleagues. We were able to mitigate this potential bias by triangulating between the participants included in our sample, but a more representative sample would further reduce this.

## 7. Conclusions

This paper reports findings from 25 interviews with stakeholders working on environmental health services in the Azraq refugee camp in Jordan. Implementing organizations had clearly defined responsibilities in the camp, but the roles of donors, the Jordanian government, refugees, and the host community were unclear. Conflicting standards and mismatched donor expectations with on-the-ground needs create challenges for providing coordinated and efficient services. Refugees’ basic needs for WaSH and waste management services are generally met, but political obstacles and inadequate resources complicate the path forward towards more sustainable services. Many participants argued that sustainability could only be achieved if refugees living in the camp were integrated into the host community. However, government policies that restrict employment opportunities for Syrians and isolate the refugees preclude this possibility.

Stakeholders working in Azraq should work to incorporate environmental, financial, and political elements of sustainability within the camp. As the Syrian conflict has persisted for over a decade and remains without a clear resolution, the assumption of refugees’ imminent departure is no longer practical. Stakeholders should instead shift to a mindset that prioritizes investing in refugees’ capacity through more participatory and development-oriented programs. Syrian refugees are not external to Jordan’s efforts to improve environmental health services and sustainability; therefore, they should be viewed as potential partners.

## Supplementary Material

SI file 1

SI file 2

**Supplementary Materials:** The following supporting information can be downloaded at: https://www.mdpi.com/article/10.3390/su16177758/s1, File S1: Interviewees; File S2: Interview guide – camp officials and stakeholders.

## Figures and Tables

**Table 1.** Summary of interviews conducted for a qualitative study on water, sanitation, hygiene, and waste management in the Azraq refugee camp, Jordan.

Characteristic	Number of Interviews (%)
**Stakeholder type**	
World Vision staff	13 (52%)
Other NGO/UN agency	6 (24%)
Refugee incentive-based volunteers	4 (16%)
Refugee community leaders	2 (8%)
**Gender**	
Male	17 (68%)
Female	8 (32%)
**Job title**	
Project or program manager	6 (24%)
WaSH specialist or engineer	5 (20%)
Community mobilizer	4 (16%)
Operations and administrative officer	4 (16%)
Hygiene promoter and behavior change specialist	3 (12%)
Waste management worker	3 (12%)

**Table 2.** Division of responsibilities for WaSH and waste management services among implementers in the Azraq refugee camp, Jordan, over time, as described in stakeholder interviews.

Topic	Responsibility	Pre-Camp Opening–6 Months	Implementer Responsible 6–24 Months	>24 Months at the Time of Study
Water supply	Initial construction	World Vision, THW *	-	-
	Water quality	World Vision, ACTED, THW *	THW *	UN Office for Project Services **
	Operation and maintenance	World Vision	World Vision	Action Contre La Faim
	Establishing WaSH committees	World Vision	World Vision	Action Contre La Faim
Sanitation	Construction	World Vision, THW *	-	-
	Operation and maintenance	World Vision	World Vision	Action Contre La Faim
	Desludging	ACTED	ACTED	UN Office of Project Services **
Hygiene	Hygiene promotion	World Vision, Relief International	WV, Relief International	Action Contre La Faim
	Hygiene kit distribution	UNHCR **	UNHCR **	UNHCR **
Waste management	Waste collection and sorting	ACTED	ACTED	World Vision
	Behavior change	ACTED	ACTED	World Vision
	Camp cleaning	ACTED	ACTED	UN Office of Project Services **, UNICEF **
Management	Incentive-based volunteer selection	CARE	CARE	CARE
	Feedback sessions	CARE	CARE	CARE
	Establishing WaSH committees	World Vision, ACTED	World Vision, ACTED	Action Contre La Faim
	WaSH sector lead	UNICEF**, UNHCR**	UNICEF**, UNHCR**	UNICEF**, UNHCR**
Other	WaSH in HCFs	International Committee of the Red Cross	International Committee of the Red Cross	International Committee of the Red Cross
	WaSH in schools	UN Office of Project Services**	UN Office of Project Services**	UN Office of Project Services**, World Vision

WaSH = water, sanitation, and hygiene; UNHCR = United Nations (UN) High Commissioner for Refugees; ACTED = Agency for Technical Cooperation and Development; THW = Bundesanstalt Technisches Hilfswerk (German Federal Agency for Technical Relief); and CARE = Cooperative for Assistance and Relief Everywhere.

* Denotes a bilateral aid agency;

** denotes a UN agency.

All other stakeholders are non-governmental organizations.

## Data Availability

The data presented in this study are available on request from the corresponding author.
