# Supplementary material for: The Evolution and Sustainability of Environmental Health Services in the Azraq Refugee Camp, Jordan: A Qualitative Study
Source: Sustainability. Author manuscript; Available in PMC 2025 Sep 23. (PMC12453110; doi:10.3390/su16177758)
Supplement: SI file 2 [file NIHMS2111176-supplement-SI_file_2.pdf]

Supplemental File S2: Interview guide – camp officials and stakeholders

Duration: 30-45 minutes
Participants: WV staff, INGO staff, camp management officials, local government officials, utility company employees, leaders of community-based organizations, and other stakeholders involved in implementation, operations and maintenance, and/or repair of WaSH and waste management programs or services in Azraq refugee camp.

Equipment: voice recorder, paper, pens

RQ1: What stakeholders are involved in WaSH and waste management services in Azraq refugee camp, and what are their respective roles?
RQ2: How frequent and effective is the coordination, communication, and sharing of information and responsibility among different stakeholders related to WaSH and waste management in Azraq?
RQ3: How has the approach to WaSH and waste management programs in Azraq changed over time?
RQ4: What are different stakeholders' perspectives of how sustainability is addressed in World Vision (and general) WaSH and waste management programs in Azraq refugee camp?

Sampling and method: A purposive sample of WaSH and waste management program stakeholders

Step 1
Before the interview the research team should provide potential participants with:
1. An understanding of why they have been asked to participate
2. Basic information about the research project as stated in the Participant Information Form
3. Clear instructions on precisely where and when the interview will take place

Step 2
Before the individual arrives for the interview, the contact researcher should ensure that:
1. The location and venue are reasonably comfortable and appropriate;
2. The recording equipment is in full working order;
3. The interview guide is available to the research team.

Step 3
At the interview, the moderator should:
1. Introduce the research team in a culturally appropriate manner;
2. Allow the individual to introduce herself/himself in a culturally appropriate manner;
3. Explain the purpose of the research, and the Participant Information Form;
4. Explain that there are no "correct" answers and that responses will be confidential;
5. Ask the participant if s/he has any questions;
6. Explain in more detail why the research team would prefer that the interview is recorded;
7. Explain the consent form and ask again if the participant has any questions or concerns;
8. If the participant consents to the interview, ask them to sign the consent form. If they do not consent, respectfully thank them for their time and move to the next interview;
9. Once the participant signs the consent form, explain that the equipment will need to be double-checked to make sure that it is recording and playing back satisfactorily;
10. When audio recording devices are turned on, record the date, time, location, researchers present, activity, and participants.

Step 4: Interview questions*

*Note: not all probes will need to be asked in every interview

RQ1: What stakeholders are involved in WaSH and waste management services in Azraq refugee camp, and what are their respective roles?

- IQ1: What is your role in WaSH and/or waste management service delivery in Azraq?
    - Probe: How long have you been in your current role? Do you have any prior experience or training that prepared you for this role?
    - Probe: What is your job description? To what extent does your job description accurately portray what you do on a day-to-day basis?
    - Probe: What resources are available to you in carrying out your responsibilities? What resources are not available?
    - Probe: Can you tell me about any monitoring or reporting responsibilities you might have?
    - Probe: What do you think is the aspect of your job at which you believe you are most effective?
    - Probe: What are the biggest challenges you face in your role? What would be needed for you to overcome these challenges?
    - Probe: What other related responsibilities do you have?
- IQ2: What other stakeholders are involved in WaSH and waste management in Azraq?
    - Probe: Who do you contact when you face a challenge related to your role?
    - Probe: Who, in your opinion, are the most important stakeholders involved in WaSH and waste management in Azraq? What makes you say that?
    - Probe: Who, in your opinion, are the most effective at fulfilling responsibilities related WaSH and waste management in Azraq? What makes you say that?
    - Probe: Are there any informal actors, institutions, or norms that affect the delivery and coordination for these services? How did they develop? What roles do they play?

RQ2: How frequent and effective is the coordination, communication, and sharing of information and responsibility among different stakeholders related to WaSH and waste management in Azraq?

- IQ1: What other actors do you work with in relation to WaSH and waste management in Azraq?
    - Probe: Could you describe your relationship with this actor?
    - Probe: In what capacity do you work with this actor?
    - Probe: Are there any actors that you do not work with who are involved with WaSH and waste management in Azraq?
- IQ2: Could you describe how communication and information sharing occurs between actors?
    - Probe: How do you communicate with other WaSH and waste management actors in Azraq?
    - Probe: How frequently do you communicate with other WaSH and waste management actors in Azraq?
    - Probe: In your opinion, is this communication effective? What makes you say that?
    - Probe: Do you ever share data or other information with other stakeholders? (If yes) How frequently? What kinds of data or information?

    - Probe: In your opinion, is the current level of communication between stakeholders appropriate?
    - Probe: What challenges have you faced in relation to communication with other WaSH and waste management actors in Azraq?
- IQ3: How is coordination among WaSH and waste management actors governed in Azraq?
    - Probe: How is responsibility for WaSH and waste management services divided among different stakeholders?
    - Probe: What formal policies, institutions, and protocols govern coordination between different actors?
    - Probe: What informal policies, institutions, and protocols govern coordination between different actors?
    - Probe: Are there any challenges related to coordination among different stakeholders?

RQ3: How has the approach to WaSH and waste management programs in Azraq changed over time?

- IQ1: (If applicable) Could you describe how WaSH and waste management were addressed in Azraq before it first opened, and in its first 6 months?
    - Probe: Who were the lead stakeholders involved at that time?
    - Probe: Could you describe how different stakeholders communicated and coordinated during that time?
    - Probe: What guiding principles, protocols, standards, or policies, if any, were most important during that time?
- IQ2: In your experience, has the approach to WaSH and waste management changed over time? In what ways?
    - Probe: Has your role changed over time? If yes, in what ways?
    - Probe: (If yes) What prompted these changes?
    - Probe: (If yes) Who was involved in the decision-making around these changes? Who was involved in the implementation of these changes?
    - Probe: What guiding principles, protocols, standards, or policies, if any, are most important for WaSH and waste management in Azraq today?
    - Probe: (If no) Do you think the approach should change? Why or why not?
    - Probe: How has the coordination between different actors changed over time, if at all?
    - Probe: What do you think is the biggest difference between the approach to WaSH and waste management now versus the approach one year ago? Compared to when Azraq first opened?
    - Probe: What, if anything, has stayed the same over time?
- IQ3: What, if any, changes do you anticipate to this approach in the future? What, if any, changes do you believe are necessary?
    - Probe: (if applicable) Why do you believe these changes will take place/ are necessary?
    - Probe: What will/would need to take place to prompt these changes?
    - Probe: Do you believe that the current divisions of responsibility among different stakeholders will change? Why? What is your opinion about this?
    - Probe: In what ways could management of these services be improved?

RQ4: What are different stakeholders' perspectives of how long-term sustainability is addressed in World Vision (and general) WaSH and waste management programs in Azraq refugee camp?

- IQ1: In your opinion, how do different stakeholders address sustainability of WaSH and waste management programs in Azraq?
  - Probe: What does "sustainability" mean to you in the context of WaSH and waste management?
  - Probe: To what extent do you believe sustainability should be a priority? Why? In what ways?
  - Probe: To what extent do you believe sustainability is a priority for different stakeholders working in Azraq?
  - Probe: In your opinion, which stakeholders are most effective at incorporating sustainability into their programs or services? Why?
  - Probe: How might different stakeholders improve their approach to sustainability in WaSH and waste management program implementation?
- IQ2: In your opinion, to what extent does World Vision incorporate sustainability into its WaSH and waste management program implementation in Azraq?
  - Probe: To what extent do you interact with World Vision staff or programs?
  - Probe: What role does World Vision play in WaSH and waste management in Azraq?
  - Probe: In your opinion, in what ways is World Vision effective at incorporating sustainability into their programs or services? What makes you say that?
  - Probe: How might World Vision improve their approach to sustainability in WaSH and waste management program implementation?

Wrap-up questions:

- In your opinion, are the needs of refugees met by current WaSH and waste management services in the camp? What makes you say that?
  - What, if anything, do you think needs to change about the current approach to WaSH and waste management in Azraq? What needs to happen to prompt that change? What makes you say that?
  - What, if anything, do you believe should be continued in the current approach to WaSH and waste management?
- What are some of the biggest obstacles to providing sustainable WaSH and waste management services in Azraq?
- In what aspects of WaSH and waste management do you think there are the biggest opportunities for improvement in Azraq?
- Do you have any recommendations about others who we should talk to about this topic?

Step 5

Towards the end of the interview, the moderator should:

1. Allow the participant the opportunity to reflect on any new insights, ideas or solutions that may have become evident during the interview; and to explore how these revelations inform action;
2. Ask the participant if 'anything else comes to mind';
3. Summarize or ask the participant(s) to summarize the conversation;
4. State that 'I don't have any more questions to ask. Do you have anything more you would like to share?';
5. Thank the participant(s);
6. Explain in detail how the participant(s) can access the research findings and any follow-up procedures;
7. Switch off the recorder to indicate that the interview is over;
8. Thank the participant(s) once again.

Step 6
Ensure any audio recordings of the day are saved and stored in a safe location.
